# From duty to dialogue: the evolution of professional trust across two generations in Mauritius

**DOI:** 10.3389/fmed.2026.1934302

**Published:** 2026-09-23

**Authors:** Mohamed Al-Eraky, Sumera Keenoo

**Affiliations:** 1 Department of Medical Education at the College of Medicine and Health Sciences, Arabian Gulf University, Manama, Bahrain; 2 Department of Medicine, Faculty of Medicine and Health Sciences, University of Mauritius, Réduit, Mauritius

**Keywords:** African context, generations, glocalization, health professions education (HPE), medical professionalism, patient expectations, perspectives, professional identity formation (PIF)

## Abstract

**Background:**

Medical professionalism is a core component of health professions education (HPE), yet prevailing frameworks have largely been developed from provider perspectives and within Western contexts. The perspectives of patients and communities, particularly in African and multicultural settings, remain underrepresented. In addition, little is known about how generational differences shape expectations of professional conduct in healthcare.

**Methods:**

This qualitative narrative inquiry investigated perceptions of medical professionalism among 32 participants in Mauritius, including 16 from Generation Z and 16 from Generation Y. Data were collected through individual narrative interviews guided by six flexible prompts: (1) an instance when a healthcare worker exceeded expectations; (2) a moment of uncertainty or discomfort in a healthcare environment; (3) a time they observed or experienced unprofessional behaviour; (4) what stood out most to them; (5) their interpretation of what it means to be a professional doctor; and (6) how their personal values influenced their expectations of doctors. The prompts served as flexible narrative guides rather than a rigid interview schedule. Participants were encouraged to develop their narratives freely and in their own sequence, with follow-up probes used where appropriate to clarify meanings, elicit further detail, and deepen their accounts. This approach provided consistency in the broad areas explored across participants while remaining aligned with narrative inquiry principles by privileging participants' experiential accounts and meaning-making. Interviews were conducted in English, French, or Mauritian Creole and transcribed verbatim. Ethical approval was secured from the National Ethics Committee of the Republic of Mauritius (Protocol MHC/CT/NETH/2025/A V2).

**Results:**

Nine themes were identified. Six themes: Humanised Care, Communication and Information Sharing, Respect and Dignity, Ethical Integrity and Trustworthiness, Accountability and Consequences, and Clinical Competence were strongly represented across both generations, forming a shared relational moral core consistent with Ubuntu-informed understandings of professionalism. Three themes showed significant generational differences. References to consent, autonomy, cultural sensitivity, and partnership-based communication were prominent in most Generation Z interviews. In contrast, Generation Y participants emphasised professional duty, vocation, moral responsibility, and stewardship more frequently. The most distinctive finding was the prominence of Duty, Vocation, and Moral Obligation among Generation Y participants (9/16) and their complete absence from Generation Z narratives (0/16). These differences suggest contrasting mechanisms through which professional trust is established and maintained.

**Discussion:**

These differences seem to reflect varying social environments rather than a decline in professional values. Generation Y grew up within more hierarchical healthcare relationships, where trust was primarily established through professional authority and moral obligation. In contrast, Generation Z has been raised in a digitally connected, rights-conscious environment that emphasises the active construction of trust through dialogue, transparency, and participation. Thus, medical professionalism in Mauritius can be understood as a fundamentally relational moral practice rooted in dignity, empathy, integrity, accountability, and competence, which serve as core values that resonate across generations, even as the preferred methods for fostering professional trust vary by cohort. Rather than signalling a shift from relationality to autonomy, these findings indicate a transition between different forms of relational professionalism. This study offers community-based insights to inform the development of culturally responsive and generationally sensitive professionalism curricula, particularly in African and other multicultural healthcare contexts.

## 1 Introduction

Medical professionalism is a concept widely integrated into health professions education (HPE) (1) yet remains critically underexplored as a generationally and culturally situated construct. Established frameworks, such as the ABIM Physician Charter (2), CanMEDS, and the General Medical Council standards, articulate professional obligations primarily through the lenses of individual autonomy, self-regulation, and competency-based conduct (3–6). These frameworks display two often-overlooked characteristics: their development within North American and European institutional contexts, and their creation by clinicians and educators rather than by the wider community whose generational composition is itself rapidly changing.

Professionalism is not a static concept; it is socially negotiated and historically evolving, continually reshaped through interactions between healthcare professionals and the cohorts they serve (7, 8). Successive generations, however, are notably absent from the frameworks that guide HPE curricula. In African and postcolonial contexts, this absence is not merely incidental but structural. Western professionalism frameworks embed assumptions about individual autonomy, professional authority, and doctor-patient relationship dynamics that may conflict with communal relational ethics, Ubuntu-informed moral vocabularies, and postcolonial power dynamics that shape healthcare across much of Africa (9–11). A genuinely “glocalized” HPE therefore requires not only adapting Western frameworks to African contexts but also empirically examining how successive generations within those contexts understand professionalism (12–14).

The literature reveals two critical gaps. First, generational differences in perceptions of professionalism remain largely unexamined within African and postcolonial HPE contexts. Existing generational scholarship has focused predominantly on healthcare providers and trainees (15), with comparatively little attention paid to how a wider, generationally diverse community, including students, health workers, patients, and caregivers, understands the domains that constitute professional conduct. Where community perspectives on professionalism have been studied, landmark contributions such as Mercer et al. (16), Stewart et al. (17), and Ginsburg et al. (18) have emerged almost exclusively from high-income Western settings. Their findings cannot be assumed to transfer to postcolonial, multicultural African societies in which relational ethics, communal accountability, and generational identity are configured differently.

Second, the generational reconfiguration of professional expectations itself remains uncharted in African settings. Generation Y (Millennials), who came of age under more hierarchical models of healthcare authority, have been shown in Western contexts to articulate professionalism through duty, vocational commitment, and institutional accountability (19, 20). Generation Z, by contrast, represents the first cohort socialised entirely within a digitally networked, rights-conscious environment, and has been shown in Western settings to expect transparency, bidirectional communication, and participatory decision-making rather than traditional deference to professional authority (21–24). Whether and how this generational shift, from a deference-based to a more participatory model of professional trust, is reflected within African, Ubuntu-informed communities remains wholly uncharted.

The significance of digital nativity in relation to healthcare professionalism warrants particular consideration. Prensky's (25) initial distinction between digital natives and digital immigrants has since been refined within health professions scholarship. Generation Z patients and trainees typically arrive at clinical encounters after conducting online searches for their symptoms, comparing various providers, and forming expectations regarding communication styles before their consultations (26). Twenge (27) documented that this generation displays significantly lower tolerance for opaque or paternalistic authority structures across various institutional contexts, not just in healthcare. Additionally, they report higher levels of anxiety and uncertainty, making transparent communication crucial for establishing trust. In the realm of health professions education (HPE), Chicca and Shellenbarger (28) found that Generation Z learners prefer interactive, dialogic instruction over traditional, didactic approaches, a preference that, according to the current study, extends into how these individuals assess the professionalism of their clinicians. Conversely, Generation Y, who transitioned to digital healthcare tools as adults rather than as digital natives, often interprets professionalism through the lens of vocation and institutional duty (15). This generational distinction concerning digital technology and information access provides a solid empirical basis, rather than merely anecdotal evidence, for anticipating differing expectations of professionalism between the two cohorts examined in this study.

Taken together, these two gaps, that is, the absence of community-derived African evidence on medical professionalism and the limited understanding of how professional expectations differ across generations, leave health professions educators without contextually relevant evidence to inform professionalism curricula across the African continent. Consequently, professionalism education continues to rely predominantly on Western frameworks whose applicability across successive African generations remains largely untested. Supporting this concern, Al-Rumayyan et al. (29), in a narrative overview of medical professionalism frameworks across non-Western cultures, identified only four culturally specific professionalism frameworks developed outside Western settings, representing Arabian, Chinese and Japanese contexts, with no comparable empirical framework from Africa. Their review further demonstrated that, although professionalism attributes converged around a small shared core (only three of 24 identified attributes were common across the three non-Western frameworks), substantial differences existed in how those attributes were interpreted and prioritised. This pattern of a shared moral core accompanied by culturally contingent enactments directly anticipates the conceptual structure of the present study. The present study addresses these two gaps by examining how Generation Y and Generation Z community members in Mauritius conceptualise medical professionalism within an African, multicultural and postcolonial context, thereby providing empirically grounded evidence to inform socially accountable professionalism curricula.

This study uses Mauritius to represent the African context, positioning it as a substantive site for exploring how global professionalism frameworks are understood across generations within a multicultural, postcolonial African setting (11). Mauritius is characterised by a rich tapestry of Hindu, Muslim, and Christian traditions, encompassing Creole, Indo-Mauritian, Franco-Mauritian, and Sino-Mauritian communities, creating a distinctive sociocultural landscape that reflects the multicultural and postcolonial dynamics found across much of the African continent. The healthcare system, comprising both public and private sectors, is shaped by postcolonial institutional dynamics common to many African health systems. At the same time, the population itself is generationally stratified, with markedly different degrees of exposure to digital, rights-based, and communal moral frameworks across cohorts. These factors render Mauritius an empirically rich African context for examining how Generation Y and Generation Z differ in their understanding of professional conduct.

The conceptual lens of Ubuntu philosophy, denoted by the phrase “Umuntu ngumuntu ngabantu,” translates to “a person is a person through other persons.” This philosophy serves as the moral framework for understanding professional legitimacy, emphasising relational humanness over mere technical credentialing or regulatory compliance (30, 31). Building on the methodological groundwork established by Al-Eraky et al. (32) through the Four-Gates model, this study posits that culture significantly influences how professional values are manifested, rather than merely their existence.

This investigation focuses specifically on how Generation Y/Millennials (born approximately 1981−1996, *n* = 16) and Generation Z (born approximately 1997−2012, *n* = 16) perceive medical professionalism in Mauritius through the lens of narrative inquiry. Participants were drawn from a generationally diverse community, including students, health workers, and patients, rather than exclusively from patients, reflecting the study's primary interest in generational, rather than purely clinical, encounter perspectives. Generational categories are treated as dynamic analytical lenses rather than fixed cohort traits, enabling a nuanced understanding of age-influenced moral interpretations, social experience, and evolving perspectives on the doctor-patient relationship, and explicitly guarding against generational “othering” (33). The analysis followed supervisory guidance to proceed inductively, organising findings by age group before exploring the underlying reasons for any observed differences (Al-Eraky, personal communication, March 2026).

In doing so, this study responds to the calls for evidence generated from African communities to inform professionalism education across the continent (11, 34, 35). Additionally, it contributes to the decolonisation of professionalism frameworks by emphasising the moral vocabularies of community participants, including patients, caregivers, students, and health workers (36). The findings from this research provide evidence-based guidance for developing glocalized Health Professions Education (HPE) curricula that encompass both enduring professional values and the evolving social expectations of diverse generational patient communities.

## 2 Materials and methods

### 2.1 Study design

This study utilised a qualitative design grounded in narrative inquiry. This approach is based on the idea that professionalism, as a lived social practice, is best understood through participants' accounts of meaningful experiences rather than through surveys or standardised assessments (37). Monrouxe (38, 39) highlighted that the construction of narrative identity is central to professional socialisation; narratives of healthcare encounters serve as acts of meaning-making through which participants express what is significant to them regarding professional conduct. Narrative inquiry is epistemologically well suited to examining how different social groups, including generational cohorts, develop and articulate their expectations of professional practice (40). Hollway and Jefferson (41) posited that narrative methods bring to light the emotional and experiential aspects of social phenomena that structured instruments often overlook. This perspective is especially pertinent when exploring culturally ingrained expectations concerning professional dignity and respect.

### 2.2 Setting and participants

The study was conducted in Mauritius, which reflects the African context through its multicultural composition, postcolonial heritage, and role at the intersection of local traditions and global healthcare discussions. Mauritius's specific configuration of ethnic and religious diversity, mixed public-private healthcare provision, and postcolonial institutional structures reflects dynamics shared across many African health systems, providing a substantive basis for examining how global professionalism frameworks are interpreted and renegotiated within African postcolonial contexts (11, 14).

Participants were recruited and interviewed between 12 May and 7 November 2025 using purposive sampling to obtain information-rich narratives from two predefined generational cohorts: Generation Z (born approximately 1997–2012) and Generation Y/Millennials (born approximately 1981–1996). Recruitment was undertaken across Mauritius through community and healthcare networks to capture a broad range of experiences and perspectives on medical professionalism. Eligible participants were adults belonging to either generational cohort who were able to provide informed consent and were willing to share their personal experiences and perceptions of healthcare encounters. Individuals who did not meet the generational criteria or who were unable to provide informed consent were excluded. Before each interview, participants received written and verbal information about the study, provided written informed consent, and were informed that participation was voluntary and that they could withdraw at any stage without consequence. Participant confidentiality was protected through anonymisation and secure data management procedures in accordance with the approved ethics protocol. A purposive sampling strategy was used to recruit 32 participants comprising 16 Generation Z participants and 16 Generation Y participants. Sampling sought to maximise diversity of gender and lived healthcare experiences within each cohort rather than statistical representativeness. The final sample comprised 16 women (50.0%) and 16 men (50.0%). Participant characteristics are presented in Table 1. Both cohorts included participants with direct experience of healthcare as patients, together with individuals who were healthcare workers, students or caregivers, thereby ensuring a range of perspectives on medical professionalism.

**Table 1:** Participant characteristics by generation and gender (*N* = 32).

Generation		Gen Y (Millennials)	Gen Z	Total
Year of Birth		1981−1996	1997–2012	
Technology Affiliation		*Digital Pioneers*	*Digital Natives*	
Gender	Females	n. 5 (31%)	n. 11 (69%)	16 Female Participants
	Males	n. 11 (69%)	n. 5 (31%)	16 Male Participants
Healthcare standpoint^a^	Patient	14 (87.5%)	11 (68.8%)	25 (78.1%)
	Health worker	3 (18.8%)	4 (25.0%)	7 (21.9%)
	Caregiver	8 (50.0%)	3 (18.8%)	11 (34.4%)
**TOTAL**		16 Gen Y Participants	16 Gen Z Participants	32 Participants

a Participants could occupy more than one healthcare standpoint (e.g., patient and caregiver); therefore, totals exceed 100% in this section.

The sample size was determined based on the principle of information power (42), rather than following a fixed numerical target. Recruitment continued until sufficient information power was achieved and no substantively new themes or subthemes emerged during analysis of the final interviews within each generational cohort. Gender diversity was maintained across both cohorts. Ultimately, the study included 32 participants, with 16 from each generational cohort. This number was considered sufficient to provide the necessary informational power for the study's specific aim: a focused generational comparison within a defined cultural context. This approach aligns with established guidelines for purposive sampling in qualitative research within healthcare professional education (43). The study does not claim generalizability; rather, the goal is to achieve analytical transferability of insights, not population-level representativeness.

### 2.3 Data collection

Data were collected through individual narrative interviews guided by six flexible prompts (1): an instance when a healthcare worker exceeded expectations; (2) a moment of uncertainty or discomfort in a healthcare environment; (3) a time they observed or experienced unprofessional behaviour; (4) what stood out most to them; (5) their interpretation of what it means to be a professional doctor; and (6) how their personal values influenced their expectations of doctors. The interviews were conducted in English, French, or Mauritian Creole, transcribed verbatim, and stored as.docx files, which serve as the primary data source.

### 2.4 Data analysis

Data were analysed using reflexive thematic analysis (43, 44). Analysis proceeded inductively through six phases: familiarisation, initial coding, theme development, theme review, theme definition, and write-up. Consistent with the methodological principle that generational comparison should structure but not predetermine analysis (33), all 32 transcripts were coded without generational assumptions before clustering by generation and constructing cross-generational comparisons. Analytic memos were maintained throughout. A data audit trail documenting all corrections is available in Supplementary Materials. Reporting of this study followed the Consolidated Criteria for Reporting Qualitative Research (COREQ) checklist (45), which is provided in Supplementary File S2.

### 2.5 Ethical considerations

This study received full ethical clearance from the National Ethics Committee (*N*EC) of the Republic of Mauritius, Ministry of Health and Wellness (Protocol reference: MHC/CT/NETH/2025/A V2; NEC Decision: 09 May 2025; Notification: 21 May 2025). The project title registered with the NEC was “What Makes a Professional Doctor in the African Context? Patient Perspectives.” All participants provided written informed consent before their involvement, in accordance with the Declaration of Helsinki (46). Confidentiality was ensured through a sequential numbering system for participants (1–32); no personally identifiable information is included in any published outputs. Data collection was limited to interview narratives, in accordance with NEC Condition (i). Ethical safeguards included the participant's right to withdraw at any time without consequences, secure data storage with password protection, and the anonymisation of all quotations utilised in analysis and reporting. The NEC clearance letter is included as Supplementary File S1.

## 3 Results

### 3.1 Participant characteristics

The final analytic sample comprised 32 participants equally divided between Generation Z (*n* = 16, 50.0%) and Generation Y (*n* = 16, 50.0%). Women constituted 68.8% of Generation Z (*n* = 11) and 31.2% of Generation Y (*n* = 5; total: 16 female, 50.0%; 16 male, 50.0%). Full characteristics appear in Table 1.

The demographic chart for gender by generation is provided as Supplementary Figure S1 at the end of this manuscript. Full demographic data are reported in Table 1, allowing the conceptual model (Figure 1) to serve as the manuscript's primary visual contribution.

**Figure 1:**
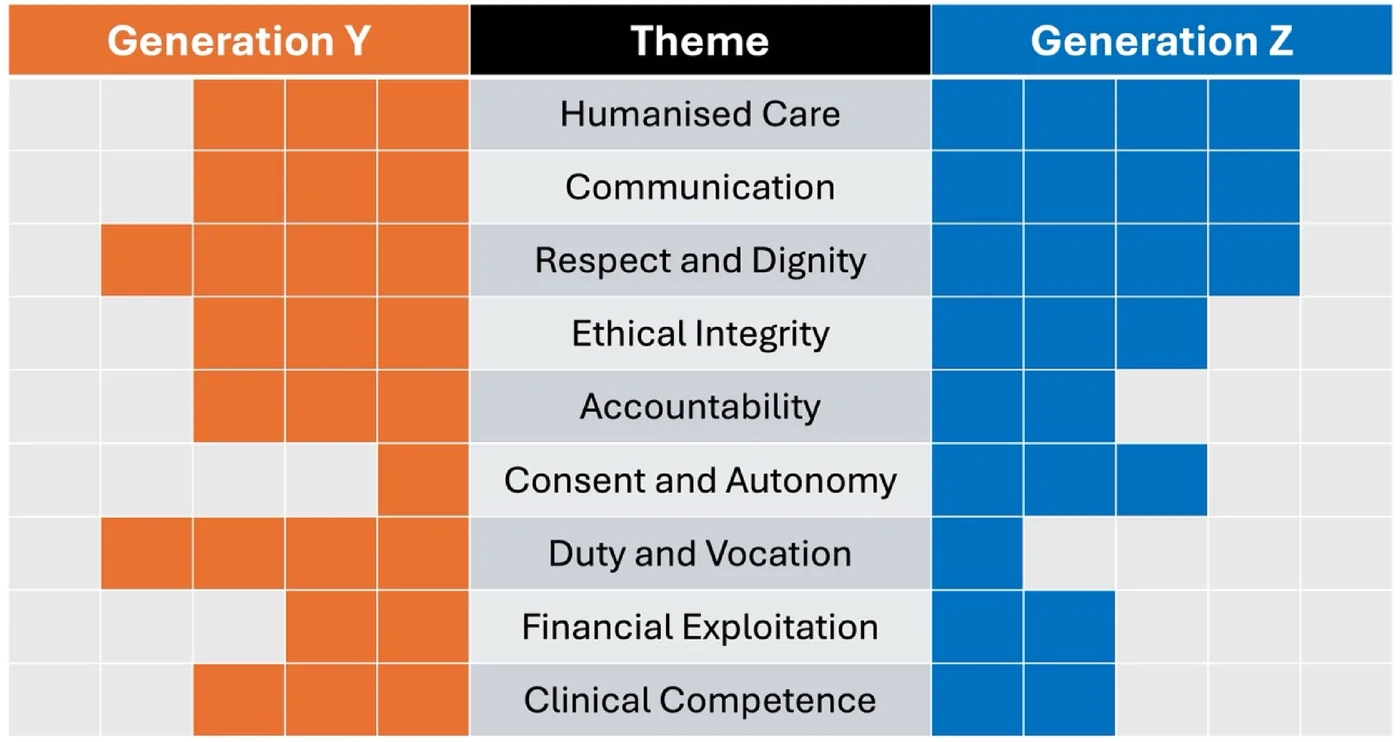
Conceptual model of the relational moral core and its generational enactment in a Mauritian African context: implications for glocalized health professions education (HPE).

Note. Source: corrected definitive dataset.

### 3.2 Thematic findings

Reflexive thematic analysis revealed nine themes. Six of these were prominently expressed by both generations, indicating a shared moral foundation. The remaining three themes demonstrated meaningful differences in thematic salience between the generations. Table 2 presents the thematic salience matrix, while Figure 2 illustrates the salience scores.

**Table 2:** Thematic salience matrix: nine themes sorted by generation.

Theme	Shared expectation	Generation Y	Generation Z
Humanised Care	Professionalism goes beyond technical skill; patients are human beings who deserve to be treated with compassion and dignity.	Duty of care; humanity within professional vocation; compassion situated within clinical competence	Relational and communicative conduct; comfort as healing; professionalism as how the doctor makes the patient feel
Communication	Clear explanation; listening without rushing; honest information-sharing	Professional responsibility; information delivery; breaking bad news sensitively	Partnership and dialogue; being genuinely heard; non-judgmental tone
Respect and Dignity	Patients must not be humiliated, stereotyped, or dehumanised	Respectful language; equal treatment; professional courtesy regardless of background	Non-discrimination; cultural sensitivity; heightened awareness of bias and stereotyping
Ethical Integrity	Honesty; avoiding exploitation; maintaining confidentiality	Honesty as professional obligation; adherence to oath; moral stewardship	Honesty as trustworthiness; transparency; consent as ethical obligation.
Accountability	Absence of consequences for unprofessional conduct shared concern	Individual moral responsibility; formal disciplinary mechanisms	System-level critique: structural enablers of poor conduct
Consent and Autonomy	Present in both; prominence differs significantly	Doctor/family-centred model largely accepted; involvement valued but not foregrounded as right	Explicit consent, privacy, right to refuse; active patient rights discourse
Duty and Vocation	Both value reliability; the language of duty diverges sharply	Hippocratic oath; vocation; life-and-death weight of responsibility	Absent.
Financial Exploitation	Both are critical of profit-driven medicine	Systemic and structural corruption: the private sector as an institutional problem	Individual betrayal; emotional violation of personal trust
Clinical Competence	Both expect competence as a necessary but insufficient condition	Foundational pillar; must be paired with ethics, duty, and accountability	Seen as baseline; necessary but insufficient without relational care

**Figure 2:**
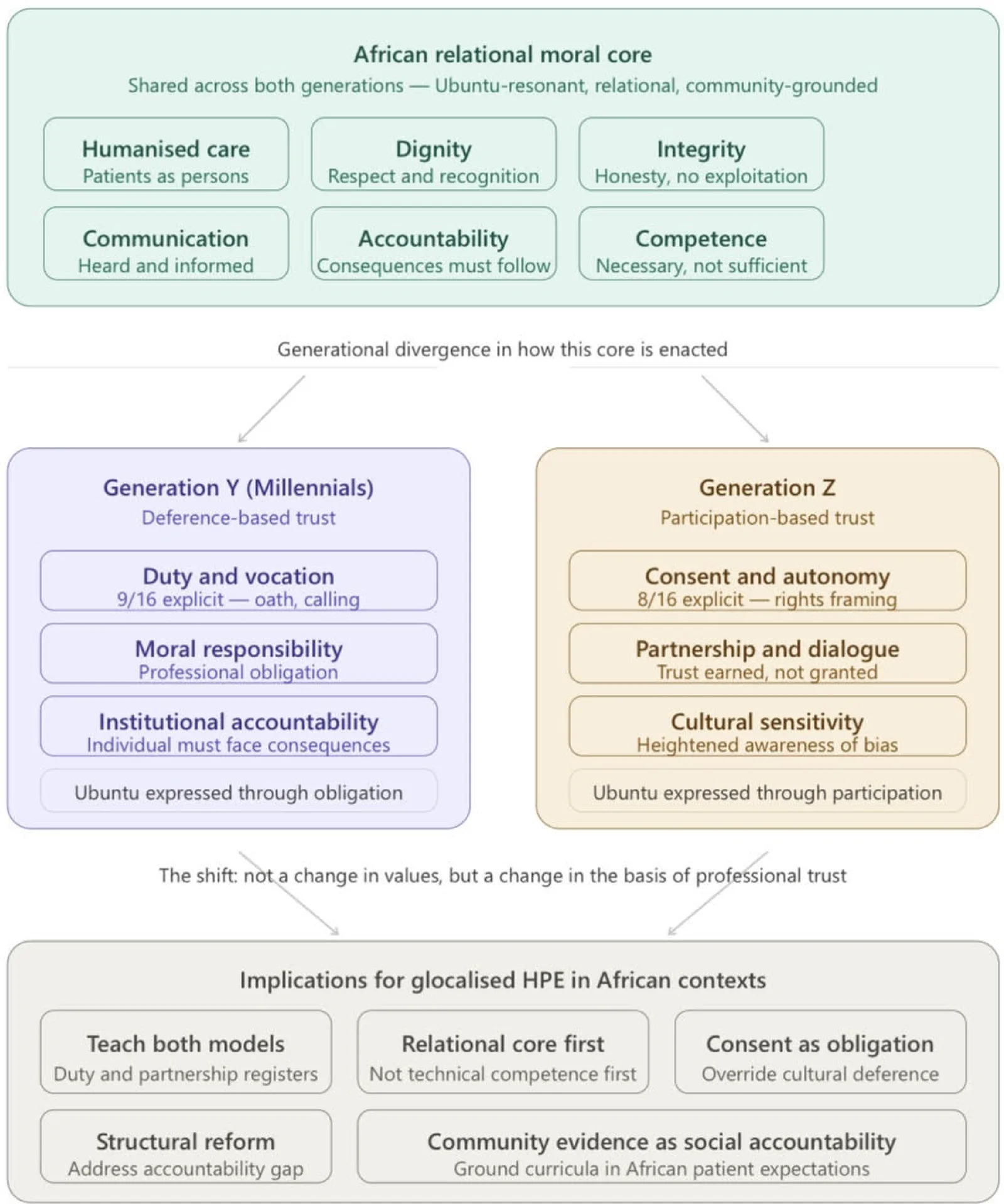
Thematic dominance on professionalism by generation.

The nine themes are presented below in order of shared salience, beginning with the six themes that constitute the relational moral core common to both generations (Themes 1−5 and 9), followed by the three themes that show meaningful generational divergence (Themes 6−8). Within each theme, Generation Y perspectives are presented first, followed by Generation Z, reflecting the chronological continuity between the two cohorts and allowing the reader to trace how each domain of professionalism is taken up, reinterpreted, or extended across generational time

#### 3.2.1 Theme 1: professionalism as humanised care

Both generations prominently express a foundational theme: professionalism extends beyond mere technical competence to encompass empathy, compassion, and the recognition of patients as whole individuals. This perspective aligns with Ubuntu relational ethics (30, 31) and is supported by Mercer et al.'s (16) findings on the therapeutic benefits of empathy, as well as Stewart et al.'s (17) concept of patient-centred medicine. Generation Y frames humanised care within the context of duty and professional obligation.

“Professionalism is not just about knowledge…it is about sacrifice, responsiveness, and empathy. Health workers must be ready to give their time and personal comfort to put the patient first.” — Participant 26, Gen Y.

Notably, Generation Z emphasised this as the foremost criterion for professionalism:

“Patients don't just remember the treatment; they remember how you made them feel.” — Participant 12, Gen Z.

“I felt awkward and uneasy. It was as if I were just a body on a bed, not a human being with feelings.” — Participant 13, Gen Z.

#### 3.2.2 Theme 2: communication and information sharing

Communication was one of the most consistently expressed expectations, aligning with the findings of Stewart et al. (17), Barry and Edgman-Levitan (47), and Levinson et al. (48). For Generation Y, communication was mainly seen as a professional responsibility that enabled adherence.

“He explained my treatment plan clearly, encouraged me, and made me feel like I could actually get better. That really improved my adherence to the healthcare plan.” — Participant 25, Gen Y.

For Generation Z, communication represented partnership and true listening, challenging paternalistic models:

“I've often experienced doctors who act like they are the only ones with knowledge. Often, they remind us that they are the professionals and that we must listen. This attitude feels very unprofessional. We deserve to be informed and involved.” — Participant 05, Gen Z.

#### 3.2.3 Theme 3: respect, dignity, and personhood

This theme highlighted the concept of universality, as nearly all 32 participants noted that dehumanising encounters are core professional failures. This aligns with Ginsburg et al.'s (18) finding that patients are especially attuned to breaches of respect, as well as Orom et al.'s (49) research on culturally marginalised populations. Generation Z exhibited a notable sensitivity to issues of discrimination and cultural bias.

“Unprofessional means when a doctor uses racist comments or judges people based on where they come from, their religion, or their background.” — Participant 15, Gen Z

#### 3.2.4 Theme 4: ethical integrity and trustworthiness

In line with Beach et al.'s (50) systematic review, both generations identified ethical integrity as fundamental to professional trust. The phrase “money-making machines,” consistently mentioned by participants in both groups, indicates a culturally shared moral vocabulary in the Mauritian context.

“They explained my condition clearly without exaggerating it. They didn't order unnecessary tests to make money. That honesty and care really surprised me.” — Participant 14, Gen Z.

“A doctor is supposed to heal, not to create harm or push a patient into unnecessary procedures for financial gain. Some private clinics turn into money-making machines.” — Participant 18, Gen Y.

#### 3.2.5 Theme 5: accountability and consequences

The near-universal expression of concern about the absence of consequences across both generations resonates powerfully with Hafferty and Franks (51) and Ginsburg et al. (18, 52). The community-level awareness of the hidden curriculum's effects has not previously been documented in the African professionalism literature:

“The lack of accountability.” — Participant 04, Gen Z

“Sadly, there were no consequences — people only talked about it, but nothing changed. That lack of responsibility really shook me.” — Participant 24, Gen Y.

#### 3.2.6 Theme 6: consent, autonomy, and patient rights

The most clearly differentiated theme between generations. Generation Z explicitly invoked patient rights such as consent, privacy, and the right to refuse, in language largely absent from Generation Y narratives. This finding extends the findings of Barry and Edgman-Levitan (47) and Elwyn et al. (53) to an African context. Critically, several Generation Z participants named the cultural norm of patient deference while simultaneously demanding that professional doctors actively override it:

“The doctor should first explain what they want to do and then ask if I agree. In Mauritius, many patients feel shy to speak up, so we often just accept what the doctor says, even if we feel uneasy. But I believe a professional doctor should always ask before touching or doing something.” — Participant 15, Gen Z.

“The patient should have the right to know and decide what happens to their body.” — Participant 16, Gen Z.

#### 3.2.7 Theme 7: duty and professional responsibility

Although thematic importance cannot be reduced to frequency alone, the complete absence of duty- and vocation-related language in the Generation Z narratives stood in sharp contrast to its prominence across 9 of 16 Generation Y narratives, making this the most distinctive generational pattern observed in this dataset. This resonates with Smith's (19) analysis of generational reframing in provider cohorts and with Cruess et al.'s (54) professional identity formation framework.

“Still believe in the oath that you should do no harm to patients. Treat every patient as if treating a family member.” — Participant 18, Gen Y.

“I wish every doctor knew that their conduct and decisions can be the difference between life and death for a patient. They must never forget the weight of that responsibility.” — Participant 24, Gen Y.

#### 3.2.8 Theme 8: financial exploitation and commercialisation

Both generations expressed criticism of profit-driven healthcare, aligning with Evetts' (7) analysis of the conflict between occupational professionalism and commercial pressures. Generation Z perceived exploitation as a personal betrayal, while Generation Y viewed it as a reflection of systemic structural corruption. This divergence in perspective can also be attributed to the differing institutional experiences detailed by Pham et al. (55).

“Some doctors see patients as bank cards or sources of money, rather than human beings who are suffering.” — Participant 10, Gen Z.

“Many doctors are like money-making machines. They lie to patients to make money. They charge fees based on patients' socioeconomic backgrounds.” — Participant 31, Gen Y.

#### 3.2.9 Theme 9: clinical competence

Both generations expected clinical competence as a necessary but insufficient condition — consistent across the international literature from Swick (6) to Stern (56):

“If the doctor doesn't even examine me, how can I trust the medicine he's giving?” — Participant 16, Gen Z

Please insert Table 3 about here.

**Table 3:** Representative quotes from both generations, organized by themes. .

Generation Y	Theme	Generation Z
Born 1981–1996 *(Digital Pioneers)*		Born 1997–2012 *(Digital Natives)*
*“Professionalism is not just about knowledge — it is about sacrifice, responsiveness, and empathy. Health workers must be ready to give their time and personal comfort to put the patient first.”* (P28/26)	**Humanised Care**	*“Patients don't just remember the treatment; they remember how you made them feel.”* (P12/07)
*“He explained my treatment plan clearly, encouraged me, and made me feel like I could actually get better. That really improved my adherence.”* (P27/25)	**Communication**	*“We deserve to be informed and involved.”* (P10/05)
*“Words can heal, or they can wound. Doctors need to be careful in the way they communicate.”* (P22/21)	**Respect and Dignity**	*“It was as if I were just a body on a bed, not a human being with feelings.”* (P45/13)
*“A doctor is supposed to heal, not to create harm or push a patient into unnecessary procedures for financial gain.”* (P17/18)	**Ethical Integrity**	*“They didn't order unnecessary tests just to make money. That honesty and care really surprised me.”* (P51/14)
*“Sadly, there were no consequences — people only talked about it, but nothing changed.”* (P26/24)	**Accountability**	*“The lack of accountability.”* (P09/04) [complete response — brevity is the finding]
*“Patients deserve full explanations before such big decisions.”* (P41/28)	**Consent and Autonomy**	*“The doctor should first explain what they want to do and then ask if I agree. A professional doctor should always ask before touching.”* (P58/15)
*“Still believe in the oath: do no harm. Treat every patient as if treating a family member.”* (P17/18)	**Duty and Vocation**	[Absent from the Generation Z corpus — this absence is the finding]
*“Many doctors are like money-making machines. They lie to patients to make money. They charge fees according to the patients' socio-economic background.”* (P59/31)	**Financial Exploitation**	*“Doctors should not act like money-making machines.”* (P51/14)
*“Professionalism means discipline in your conduct and being truly committed to giving your patients the best care possible.”* (P26/24)	**Clinical Competence**	*“If the doctor doesn't even examine me, how can I trust the medicine he's giving?”* (P63/16)

### 3.3 Cross-generational synthesis: stable core, evolving enactment

The nine themes together reveal an analytically rich finding. Both generations articulated a shared moral core, humanised care, communication, dignity, ethical integrity, accountability, and competence, and rejected purely technical definitions of professionalism. What differed was how professionalism should be enacted and on what basis professional trust should be established. Generation Y operated within a deference-based model: trust arises from duty, moral responsibility, and vocational commitment. Generation Z described a participation-based model in which trust is earned through communication, transparency, consent, and partnership. Importantly, these findings should not be interpreted as evidence that one generation values professionalism more than the other. Both cohorts expressed remarkably similar commitments to dignity, honesty, accountability, and compassionate care. The divergence concerned the social processes through which these commitments become visible and trustworthy within clinical relationships. Table 4 summarises the orientations.

**Table 4:** Overarching generational orientations toward medical professionalism.

Dimension	Generation Y	Generation Z
Basis of trust	Deference-based: Expertise, moral integrity, professional authority.	Participation-based: Dialogue, transparency, relational engagement.
Primary frame (Question)	What the doctor is duty-bound to do?	How the doctor makes the patient feel?
Patient role	Respected beneficiary: Welfare as a doctor's moral responsibility.	Active partner: Right to be heard, informed, and involved.
Communication	Professional responsibility; information delivery; clinical standards.	Partnership; genuine hearing; validation of experience.
Decision-making	Ethical authority with clear explanations; professional guidance.	Collaborative; shared; right to question or refuse.
Accountability	Individual responsibility; Consequences must follow misconduct.	System critique: Structures enabling poor conduct.
Shared understanding across both generations	Both generations considered professionaism as a blend of the following elements: competence, empathy, respect, ethical integrity, and dignity. Neither generation accepts a purely technical definition of competence of dispcline-related expertise in medicine.	

## 4 Discussion

### 4.1 Principal findings and conceptual contribution

This study makes three interconnected contributions, grounded in the foundational methodological principles established by Al-Eraky et al. (32) and Al-Eraky and Marei (57) for non-Western professionalism research. They emphasise that professionalism should be seen as a culturally embedded belief system rather than just a list of observable traits.

First, drawing on narratives from 32 participants equally divided between Generation Y (*n* = 16) and Generation Z (*n* = 16), including patients, healthcare workers, students and caregivers, this study provides one of the first empirical characterisations of generational differences in community perspectives on medical professionalism within an African context. Second, it divergence in core professional values; both generations articulated a shared relational moral core consistent with Ubuntu-informed African scholarship. Instead, the principal distinction lies in the basis and mechanism of professional trust: for Generation Y participants principally described deference-based professional trust grounded in professional authority, duty and moral integrity, whereas for Generation Z participants emphasised participation-based professional trust grounded in dialogue, transparency, relational engagement and shared decision-making. This distinction resembles the Four-Gates model identified by Al-Eraky et al. (32) in the Arabian context, reinforcing the proposition ng that culture shapes how professional values are enacted and prioritised rather than whether those values exist.

The overarching principle of the Four-Gates model, which emphasises “dealing with God,” establishes a transcendent moral framework that underpins all professional obligations. In a similar vein, data from Mauritius' Generation Y reveal a comparable moral structure, in which duty, vocation, and the Hippocratic oath serve as the foundational elements that legitimise professional authority. Meanwhile, Generation Z adopts a participation-based model that, while not entirely discarding this sense of transcendence, shifts the focus of moral legitimacy from role assignment to relational demonstration. This transition emphasises communicative performance over ontological status.

Furthermore, this study contextualises these empirical findings within a social accountability framework (58) and aligns them with the broader discourse on glocalisation in health professions education (HPE). It provides evidence-based guidance for African HPE institutions seeking to develop professionalism curricula that are both globally credible and locally responsive.

### 4.2 From deference-based trust to participation-based trust: the central generational finding

The most significant generational distinction identified in this dataset is the shift in how professional trust is established. Participants from Generation Y (9 of 16) framed professionalism through the lenses of duty, vocation, the Hippocratic oath, and moral obligation. For them, trust is deference-based and granted to doctors who clearly accept professional responsibility. In contrast, Generation Z participants (none of the 16 used language referencing duty or vocation) spoke about a participation-based model of trust, emphasising consent and patient rights. They described trust as being earned through communicative engagement in each interaction.

Existing frameworks of professionalism have often positioned autonomy and relationality as competing moral perspectives. However, the current findings reveal a different narrative. Both generations articulated a fundamentally relational understanding of professionalism. The distinction was not about the importance of relationships, but rather about how relational commitment should be expressed. For Generation Y, relational professionalism was manifested through duty, vocation, stewardship, and moral responsibility, with Ubuntu enacted through obligation. Conversely, Generation Z expressed it through dialogue, transparency, consent, and partnership, enacting it through participation.

The transition is not a movement from relationality to autonomy; rather, it reflects a shift from one form of relationality to another. In the African context, several Generation Z participants emphasised the cultural norm of patient deference, asserting that professionals must actively confront it. This represents a more significant social reorientation than the generational shifts seen in Western contexts.

Existing professionalism frameworks often position autonomy and relationality as competing moral paradigms. The present findings suggest otherwise: both generations remain fundamentally relational in orientation, and what distinguishes them is not whether relationships matter but how relational commitments are enacted and how professional trust is produced. Generation Y participants articulated a form of relational professionalism grounded in duty, stewardship, vocation, and moral obligation, in which trust arises through professional authority and institutional responsibility. Generation Z participants described a form of relational professionalism enacted through dialogue, transparency, consent, and partnership, in which trust is actively constructed rather than conferred by role alone. These findings, therefore, indicate not a movement from relationality to autonomy, but a transition between two forms of relational professionalism, a conceptual distinction that extends beyond this Mauritian dataset and offers a generalizable framework for interpreting generational change in professionalism across cultural contexts.

### 4.3 Two forms of relational professionalism

#### 4.3.1 Alignment with and extension of prior literature

The observation that Generation Z patients prioritize communication, participation, and rights-based engagement corresponds with the findings of Cecconi et al. (59) and Madanian et al. (60), who noted younger patients' preference for participatory, digitally enhanced, and transparent healthcare. However, both studies were conducted in high-income Western contexts. This study reveals that similar patterns emerge in a multicultural African Small Island Developing State (SIDS), albeit with contextually significant variations. Mauritian Generation Z participants expressed their rights discourse in contrast to a recognised cultural norm of patient deference, an aspect not addressed in Western literature, which does not engage with the same cultural framework. This contextual nuance is essential for the scholarship on glocalisation (14).

The study also finds that Generation Y patients frequently invoke duty, vocation, and the Hippocratic oath, which aligns with the observations of Smith (19) and Irvine (5). Notably, this study documents this trend among lay patients rather than physicians, suggesting that the generational reframing observed in provider literature is reflected in patient expectations, with direct implications for curricula. There is a near-universal concern about the lack of consequences for professional misconduct, echoing the arguments of Hafferty and Franks (51), who contend that institutional tolerance for misconduct systematically undermines the professional values formally taught. Similarly, Ginsburg et al. (18) found that the public expects repercussions for breaches of professionalism, which professionals themselves often rationalise as acceptable. This study contributes community-level evidence from a Mauritian African context, offering a previously undocumented perspective.

#### 4.3.2 Professionalism as relational moral practice: insights from a Mauritian African context

The relational moral core identified across both generations aligns with Ubuntu-informed conceptions of professionalism in African scholarship (30, 31) and resonates with the broader literature on cross-cultural professionalism. Jha et al. (61), in a systematic review of medical professionalism across cultures, revealed that while fundamental principles such as honesty, altruism, and competence are universally shared, the relational, contextual, and spiritual dimensions of professionalism exhibit significant variation. This variation is not merely incidental; it is constitutive, with the cultural enactment of professionalism being integral to its very meaning rather than a superficial expression of deeper universal principles.

Ubuntu, exemplified by the proverb “Umuntu ngumuntu ngabantu,” underscores that personhood is established through relationships: “I am because we are.” A doctor who examines a patient without explaining, or addresses the body without recognising the individual, neglects a fundamental professional obligation within this relational moral framework. This framework necessitates acknowledging and sustaining the patient's personhood through meaningful engagement. This is why the phrase “not just a body on a bed, not a human being” recurs among various Generation Z participants; it is not simply a stylistic complaint but a moral assertion rooted in Ubuntu ethics and articulated in patient vernacular.

Both Generation Y and Generation Z express this Ubuntu-inspired relational ethic, though in distinct ways. Generation Y conveys it through notions of duty and obligation, reflecting what Al-Eraky et al. (32) observed in the Arabian context as “dealing with others” under the encompassing theme of “dealing with God”: a relational professional responsibility rooted in transcendent moral conviction. Conversely, Generation Z articulates it in terms of partnership and rights; their relational responsibilities manifest in seeking consent, sharing information, and engaging the patient as a co-participant. This form of relational ethics does not depend on a transcendent foundation but is instead grounded in democratic recognition and dialogue.

Al-Rumayyan et al. (29), in a narrative overview of medical professionalism frameworks across non-Western cultures, identified only three structured culture-specific frameworks in the literature between 2002 and 2014, from Arabian, Chinese, and Japanese contexts and found that of 24 identified professionalism attributes across these three frameworks, only three (altruism/compassion, integrity/honesty, and accountability/commitment) were shared by all three cultures, while the majority diverged in interpretation and prioritisation. This pattern of a small universal core surrounded by substantial culturally specific divergence is precisely what the present study documents at the generational rather than cross-cultural level. Three comparative points are instructive. First, in the Arabian framework, Al-Eraky et al. (32) located self-accountability and self-motivation within an overarching “dealing with God” gate, interpreting them through the Islamic concepts of taqwa and ehtesab, professional conduct grounded in answerability to a transcendent authority rather than to peers or regulators alone. The present study's Generation Y participants articulate a structurally similar architecture without religious specificity: duty and vocation are invoked as a moral calling that transcends individual patient encounters, echoing the transcendent grounding Al-Eraky and Macdougall (62) identified in the Arabian context. Second, in the Chinese framework, Ho et al. (63) and Pan et al. (64) identified Zizhong — self-dignity and respect, as the attribute Chinese stakeholders added to Stern's Western framework, treating it as foundational rather than peripheral; this resonates with the present study's Theme 3 (Respect, Dignity, and Personhood), in which both Mauritian generations treated dignity as a non-negotiable baseline rather than an optional courtesy. Third, in the Japanese framework, Nishigori et al. (65) found that Bushido's code of the warrior, incorporating rectitude, courage, benevolence, and loyalty, converged with the Physician Charter on most attributes but diverged sharply on patient autonomy, which the Charter foregrounds and Bushido omits entirely. The present study's Generation Z participants demonstrate the inverse trajectory: autonomy and consent are foregrounded precisely because the surrounding cultural deference norm, structurally similar to what Bushido's loyalty-to-group ethic would predict, is being actively contested from within the younger generation rather than accepted as given. Together, these three comparisons demonstrate that the Mauritian generational divergence reported here is not idiosyncratic but follows a documented pattern in non-Western professionalism scholarship: culture, and in this study, generation supplies the register through which a narrow universal core is enacted, prioritised, and contested.

Birden et al. (66), in a systematic review of definitions of professionalism in medical education, found that most published definitions inadequately capture these relational, contextual, and generational dimensions. The current study contributes empirical evidence from an African patient community that precisely addresses this gap, demonstrating that professionalism in African contexts is both relationally grounded and evolving generationally, and that these two dimensions must be considered together in any comprehensive theoretical account.

Sullivan et al. (67), through a modified Delphi study conducted at the Shapiro Institute at Harvard Medical School, sought to redefine the concept of professionalism in contemporary medicine. Their consensus highlighted relational competence, the ability to authentically engage with patients across various cultural, generational, and experiential differences, as a vital aspect of professionalism that previous frameworks have often overlooked. This finding from a prestigious Western medical institution resonates with evidence from an African community presented in this study. Both data sets, derived from markedly different contexts, arrive at the same conclusion: professionalism is fundamentally relational rather than solely technical. This underscores the necessity for a clear educational focus on the relational dimensions of professional practice.

Sadeq et al. (68), in their systematic review of medical professionalism education interventions, observed significant methodological variability and limited sustainability in current educational approaches. They pinpointed the lack of contextually responsive, long-term strategies that foster meaningful professional identity formation as a critical gap in the field. This study addresses that gap from a community perspective, providing the local evidence needed for contextually relevant professionalism education. These insights are specific to the Mauritian context; whether similar trends can be found in other African settings remains an empirical question for future comparative research.

These findings contribute a new conceptual distinction to the professionalism literature: the difference between the relational moral core, what patients across both generations consistently expect, and the enactment register through which that core is expressed and legitimised in clinical encounters. The relational moral core (humanised care, dignity, integrity, communication, accountability, competence) is stable across generations. What is evolving generationally is not the core itself but the mechanism by which professional trustworthiness is demonstrated: through duty and vocation (Generation Y) or through dialogue and consent (Generation Z). This distinction, between a shared moral foundation and divergent mechanisms of enactment, is the study's primary conceptual contribution and provides a more precise account of generational change in professionalism than existing frameworks, which tend to treat generational differences as differences in values rather than in relational moral practice.

### 4.4 Implications for glocalized health professions education

#### 4.4.1 Curricular and social accountability implications

Five evidence-based implications follow for HPE curriculum design in Mauritius and across comparable African contexts, each grounded in both the present data and the existing HPE scholarship. First, professionalism curricula must teach both the deference-based and participation-based models of professional trust simultaneously. Al-Eraky et al. (69) demonstrated that culture-sensitive faculty development, using locally developed vignettes embedded in recognisable clinical and social contexts, is more effective than imported curricula in developing authentic professional identity. The same principle applies: professionalism curricula in Mauritius and similar African contexts must be developed in collaboration with the communities they serve. These curricula should incorporate both the duty-vocation register valued by Generation Y patients and the dialogue-partnership register that Generation Z demands. A curriculum focused solely on one register will not adequately prepare graduates for the generational diversity of their patient communities.

Botha and Crafford (70) demonstrated that social responsiveness in African health professions education (HPE) requires graduates who can navigate diverse community contexts and adapt their professional conduct accordingly. This aligns with the dual-register competency identified in the present study.

Additionally, the relational moral core should be the primary framework of the curriculum, rather than merely a supplementary skill. Al-Eraky and Marei (57) argued that professionalism cannot be reduced to a list of attributes because it represents a belief system. Teaching professionalism effectively involves engaging learners with the moral convictions that drive professional behaviour, rather than focusing solely on behavioural outcomes.

In African contexts, where Ubuntu-influenced relational ethics are already part of patient moral vocabularies, curricula that highlight relational humanity as the core of professional identity will be more culturally relevant and pedagogically effective than those that consider communication and empathy as add-on competencies.

Furthermore, teaching patient autonomy and consent must be approached as culturally urgent obligations, rather than as Western imports. Guraya et al. (71) showed that digital professionalism and patient engagement increasingly require healthcare professionals to involve patients as active participants rather than passive recipients. This shift is reflected in the Generation Z data from the present study. In African contexts, where cultural deference norms pose specific risks of patient disempowerment, teaching consent as an active professional responsibility, one that may require overriding social norms rather than simply complying with legal requirements, is an imperative in African HPE. This need is not adequately addressed by Western autonomy frameworks, which assume patients are already aware of their rights.

Lastly, the accountability gap requires institutional reform alongside curricular changes. Hafferty and Franks (51) demonstrated that the hidden curriculum is as influential as explicit instruction. When students observe that unprofessional conduct carries no institutional consequences throughout their training, the formal teaching of professionalism is systematically undermined. The widespread community awareness of the lack of consequences documented in this study, across both generational cohorts, indicates that this is not merely a student observation but a community-level experience that undermines professional trust across generations. Jha et al. (61) argued that the cross-cultural challenge in professionalism education lies not in identifying universal principles. Still, their contextually appropriate enactment requires institutional structures that make professional values visible and consequential. Fifth, African HPE institutions must ground professionalism education in locally generated community evidence to fulfil the social accountability mandate (58, 72). The WHO (73) Transforming Health Professions Education framework identifies social accountability as a non-negotiable principle for 21st-century HPE reform. The present study offers a framework for generating evidence through narrative inquiry involving diverse community members, which is analysed inductively. This approach yields findings that are both empirically grounded and theoretically consistent with the broader literature on African relational ethics, non-Western professionalism, and glocalised health professions education (HPE).

Additionally, there are implications for professional identity and well-being. Sattar et al. (74) demonstrated, using structural equation modelling, that professionalism serves as a buffer against burnout among medical students and is positively associated with empathy through adaptive coping strategies. This finding is particularly relevant to the current study's data from Generation Z: the focus on communicative partnership, psychological safety, and bidirectional engagement may indicate not just a preference for different professional styles but also a well-being-oriented approach to professionalism, viewing it as authentically relational rather than merely compliant. This finding aligns with calls to nurture professionalism in a culturally responsive way across diverse educational settings (75).

HPE programs that teach professionalism as a dynamic, relational, and psychologically sustaining practice, rather than a set of externally imposed obligations, are more likely to cultivate graduates who genuinely embody professional values, rather than merely perform them.

#### 4.4.2 Implications for glocalized health professions education: A synthesis

The special issue in which this article appears is explicitly framed around glocalisation as a “critical imperative” for HPE, the argument that globally credible educational standards must be actively adapted to local sociocultural realities rather than exported intact. The present study provides empirical evidence for precisely this imperative from the community side of the educational encounter. Glocalized professionalism education in Mauritius and comparable African settings requires integrating three elements that current global frameworks do not adequately provide. The global elements, competence, ethical integrity, accountability, and commitment to patient welfare, are affirmed by the present data. Both generations hold these values, and neither accepts a purely technical or regulatory definition of professionalism. Global frameworks can and should provide the normative architecture that governs local professional conduct (2, 4, 76). The local African elements- Ubuntu relational ethics (“I am because we are”); communal accountability; the specific cultural dynamics of patient deference toward professional authority; the postcolonial dimension of the public-private healthcare boundary; and the generationally stratified moral vocabulary of professional trust- cannot be derived from global frameworks. They must be empirically discovered from community engagement of precisely the kind this study models. Boelen and Woollard (58) argued that social accountability is not optional for HPE institutions; it is the condition of their professional legitimacy. This study fulfils that condition by providing the community-grounded evidence that a socially accountable curriculum design requires. The curriculum implications of this glocalisation argument are specific. Communication training must be designed for both the duty-based register that Generation Y patients expect and the participatory, consent-centred register that Generation Z patients increasingly demand. Patient partnership must be taught not as a courtesy add-on but as a substantive professional obligation with specific relevance to African contexts where cultural deference creates structural risks of patient disempowerment. Consent education must address the specific African context in which “many patients feel shy to speak up” (Participant 15, Gen Z), emphasising active consent-seeking rather than passive compliance with requests. Community-informed professionalism assessment must incorporate evidence of the kind generated by this study, extending beyond the provider-generated, instrument-derived definitions that dominate existing assessment frameworks (77).

#### 4.4.3 Professional identity formation implications

The PIF literature (54, 78, 79) suggests that generational variation in how professional values are expressed may reflect different communities of practice and different socialisation histories. Generation Z participants are forming professional expectations in a digitally connected, rights-aware, and increasingly sceptical social environment. HPE programmes that model professional identity exclusively through duty, hierarchy, and vocational self-sacrifice will increasingly fail to speak to this generation's emerging professional self-understanding. Steinert et al.'s (80) evidence that faculty development must address the hidden curriculum and role modelling alongside formal instruction is directly relevant. If clinical educators and supervisors model paternalistic communication and reward deference over dialogue, the hidden curriculum will undermine any formal curriculum elements that teach patient partnership.

### 4.5 Strengths and limitations

This study's strengths include its focus on generational and community perspectives in an underrepresented African context, its inductive narrative inquiry design, its incorporation of the complete verified dataset, and its rich narrative accounts, which enabled an in-depth exploration of how professional trust is understood across generations. Several limitations should nonetheless be acknowledged. First, the study employed purposive sampling within a single African national context (*n* = 32); therefore, the findings should be interpreted with appropriate caution when considering their transferability to other African settings, even though Mauritius provides a valuable multicultural and postcolonial context through which broader African dynamics may be explored. Second, participants were recruited and interviewed between 12 May and 7 November 2025, reducing prolonged recall intervals; however, narrative interviews remain retrospective accounts and are therefore susceptible to recall bias and social desirability bias, particularly when recounting experiences of unprofessional conduct. Third, while Mauritius's multicultural and postcolonial character offers a valuable setting for examining how global professionalism frameworks are interpreted and renegotiated across the African continent, future research should extend this analysis to additional African contexts to corroborate and enrich the present findings. Finally, the primary coding was undertaken by the principal investigator without independent inter-rater verification. Nevertheless, reflexive memo-writing, iterative engagement with the data, and regular supervisory discussions were used throughout the analytic process to challenge assumptions, enhance reflexivity, and strengthen the credibility of the interpretations. Future research should include larger multisite studies across diverse African contexts and incorporate multiple analysts to further strengthen analytical rigour.

### 4.6 Methodological reflexivity

The researcher, a medical educator from Mauritius, possesses extensive experience in health professions education, clinical teaching, and ENT practice. Their prior engagement in professionalism scholarship, including a published reflective essay on the path to professionalism (81), may have predisposed interpretation toward relational and Ubuntu-informed readings of the data. To mitigate this risk, reflexive memos were maintained throughout data collection and analysis. Discussions with the second author, Prof. Al-Eraky, offered an invaluable external check. His foundational methodological guidance promoted an open-minded approach to the interviews, initially categorising findings by age group before exploring the reasons behind any observed differences (Al-Eraky, personal communication, March 2026), and helped orient the analysis toward emergence rather than confirmation. Coding was conducted inductively across all 32 transcripts before applying any generational groupings. The researcher intentionally set aside preconceived notions regarding generational differences, such as Generation Z's digital orientation and Generation Y's duty-based mindset, and critically examined these assumptions throughout the analysis. Generational categories served as organisational tools for comparison rather than explanatory frameworks, in line with Jauregui et al.'s (33) caution against generational “othering” and consistent with the guidance received from the supervisory author. This reflexive positioning is reported here, in the Discussion, rather than in the Methods, because its primary purpose is to support the reader's critical interpretation of the findings reported above, rather than to describe a procedural step in data collection.

## 5 Conclusion

Medical professionalism in this Mauritian African context is best understood not as a fixed set of competencies but as a relational moral practice grounded in dignity, empathy, integrity, accountability, and competence. Although these values remain remarkably stable across generations, the mechanisms through which professional trust is established are evolving. Generation Y tends to place trust in professional duty, vocation, and moral stewardship, whereas Generation Z expects trust to be actively built through dialogue, transparency, consent, and partnership. These findings suggest not a transition from relationality to autonomy but a shift between two forms of relational professionalism. Glocalized health professions education should therefore preserve enduring moral commitments while preparing future clinicians to enact professionalism through culturally responsive, generationally sensitive forms of trust.

## Data Availability

The raw data supporting the conclusions of this article will be made available by the authors, without undue reservation.
