# Supplementary material for: From duty to dialogue: the evolution of professional trust across two generations in Mauritius
Source: Front Med (Lausanne). 2026 Sep 23;13:1934302. doi: 10.3389/fmed.2026.1934302 (PMC13642010; doi:10.3389/fmed.2026.1934302)
Supplement: Supplementary file 1 [file Datasheet1.pdf]

REPUBLIC OF MAURITIUS
MINISTRY OF HEALTH AND WELLNESS

MHC/CT/NETH/2025/A V2 **21 May 2025**

Dr Bibi Sumera KEENOO
93A, Ducray Lane
Jerningham Street
Curepipe

Dear Madam,

Request for Ethical Clearance

Please refer to your correspondence regarding the above-mentioned subject and be informed that your application for Ethical Clearance in respect of the project entitled ***"What makes a Professional Doctor in the African context? Patient Perspectives"*** has been examined at the level of the Ministry.

2. This is to inform you that the National Ethics Committee has given Ethical Clearance for the above project, subject to list of conditions annexed.

Yours faithfully,

Dr V. T. Mohabeer
for Acting Senior Chief Executive

REPUBLIC OF MAURITIUS
MINISTRY OF HEALTH AND WELLNESS

The National Ethics Committee
Decision

Project Protocol : MHC/CT/NETH/2025/A V2

Applicant : **Dr Bibi Sumera KEENOO**

Project Title : **What makes a Professional Doctor in the African context? Patient Perspectives**

The National Ethics Committee Meeting held on 09 May 2025 has

Awarded Ethical Clearance

to the above project proposal.

You are also requested to: -

(a) submit a Progress Report every month;

(b) notify the Ethical Committee of any amendment of recruitment of material or of consent form, or of information to be submitted to the research participants;

(c) report to the Ethical Committee any serious or unexpected, unforeseen circumstances;

(d) report to the Ethical Committee termination of the project;

(e) provide relevant information to the Ethical Committee for ongoing review;

(f) submit a copy of the project, with final results and outcomes, to the National Ethics Committee, prior to publishing of the project;

(g) ensure that confidentiality is respected throughout the project;

(h) ensure that your research is in strict compliance with the Quarantine Act 2020; and

(i) note that access would be granted for collection of data only and not patient files.

Yours faithfully,

Dr V. T. Mohabeer
for Acting Senior Chief Executive
